# Dr. Ellis W. Storms

**Published:** 1918-03

**Authors:** 


**Dr. Ellis W. Storms,** University of Buffalo, 1900, died in Falconer, age 49.

